# Supplementary material for: The Psychometric Properties of the DASS-21 and Its Association with Problematic Internet Use among Chinese College Freshmen
Source: Healthcare (Basel). 2023 Feb 27;11(5):700. doi: 10.3390/healthcare11050700 (PMC10000485; doi:10.3390/healthcare11050700)
Supplement: Supplementary file 1 [file healthcare-11-00700-s001.zip › healthcare-2124403-supplementary.pdf]

We conducted Exploratory Factor Analysis (EFA) on Sample 1 and Sample 2 separately, utilizing the principal axis factoring extraction method and Promax rotation. The Scree plots indicate that the factor structure of the DASS-21 may deviate from the initially anticipated three-factor structure. This is evident from Figures S1 and S2 which suggests that a two-factor solution is more likely to be extracted. Additionally, the utilization of the three-factor extraction fixing method has revealed that several items in Tables S1 and S2 exhibit cross-loadings, meaning that the items belong to multiple factors simultaneously.

Table S1. Factor loadings from Exploratory Factor Analysis of the DASS-21 for Sample 1 (October 2020)

	Factor			
	**1**	**2**	**3**	**Uniqueness**
Item 9	0.689			0.581
Item 11	0.667			0.510
Item 8	0.572			0.622
Item 12	0.522			0.479
Item 5	0.521			0.652
Item 1	0.498			0.736
Item 2	0.496			0.751
Item 13	0.406		0.321	0.451
Item 3	0.357			0.744
Item 18	0.327			0.774
Item 6	0.325			0.805
Item 17		0.786		0.346
Item 16		0.717		0.472
Item 10		0.659		0.491
Item 21		0.655		0.542
Item 15		0.327		0.582
Item 19			0.579	0.673
Item 4			0.574	0.688
Item 7			0.406	0.802
Item 20			0.370	0.674
Item 14				0.815

Note. The 'Minimum residual' extraction method was used in combination with a 'Promax' rotation

Table S2. Factor loadings from Exploratory Factor Analysis for Sample 2 (October 2022)

	Factor			
	1	2	3	Uniqueness
Item 21	0.978			0.284
Item 17	0.941			0.266
Item 16	0.633			0.442
Item 10	0.554	0.467		0.410
Item 15	0.476		0.411	0.378
Item 13	0.372	0.363		0.349
Item 18				0.554
Item 9		0.910		0.436
Item 5		0.619		0.520
Item 11		0.565		0.362
Item 8		0.530		0.495
Item 14		0.506		0.646
Item 12		0.425		0.376
Item 3				0.548
Item 7			0.675	0.602
Item 4			0.637	0.553
Item 2			0.569	0.610
Item 19	0.316		0.558	0.453
Item 20	0.386		0.390	0.452
Item 6		0.330	0.361	0.529
DASS21_1			0.352	0.654

Note. The 'Principal axis factoring' extraction method was used in combination with a 'Promax' rotation

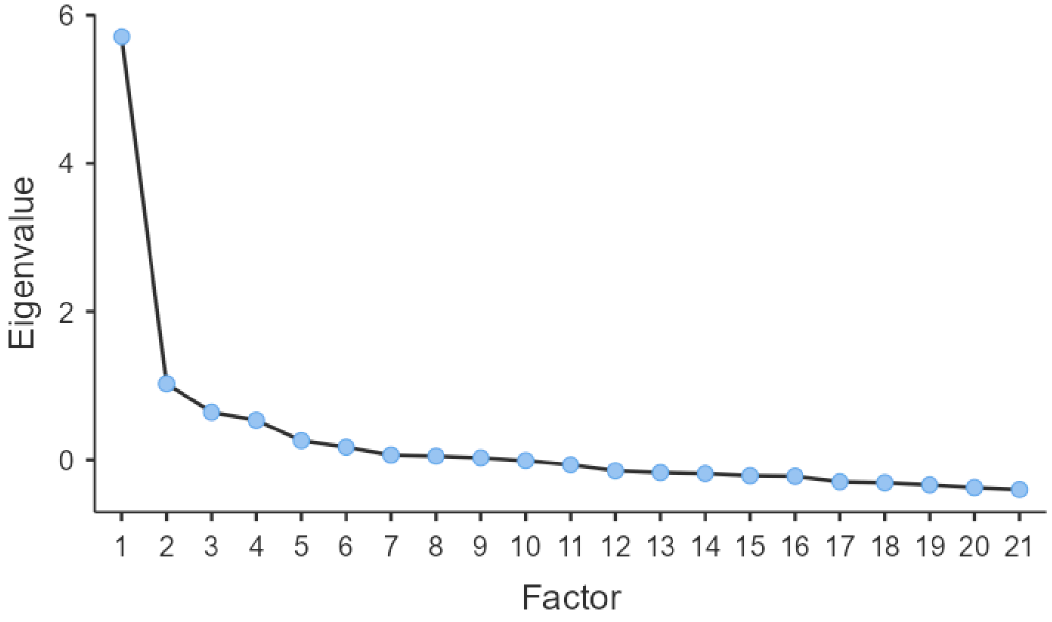

Figure S1. Scree plot from Exploratory Factor Analysis of the DASS-21 for Sample 1 (October 2020)

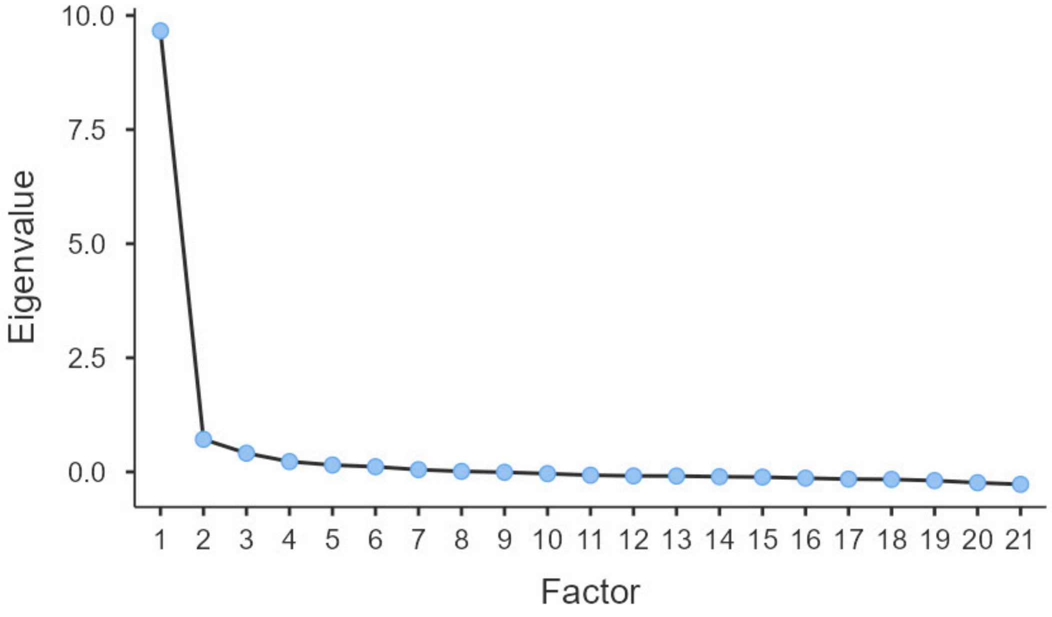

Figure S2. Scree plot from Exploratory Factor Analysis of the DASS-21 for Sample 2 (October 2022)
